# Investigation of Grignard Reagent as an Advanced Base for Aza-Claisen Rearrangement

**DOI:** 10.3390/molecules24244597

**Published:** 2019-12-16

**Authors:** Bo Reum Song, Min Woo Ha, Donghwan Kim, Chanin Park, Keun Woo Lee, Seung-Mann Paek

**Affiliations:** 1 College of Pharmacy, Research Institute of Pharmaceutical Sciences, Gyeongsang National University, Jinju daero, Jinju, Gyeongnam 52828, Korea; qhfma2005@naver.com (B.R.S.); beneminu_@naver.com (M.W.H.); 2 Division of Life Science, Division of Applied Life Science (BK21 Plus), Research Institute of Natural Science, Gyeongsang National University, Jinju 52828, Korea; donghwan0651@gmail.com (D.K.); chaninpark0806@gmail.com (C.P.); kwlee@gnu.ac.kr (K.W.L.)

**Keywords:** aza-Claisen rearrangement, Grignard reagent, ring expansion, base, molecular dynamics

## Abstract

Employing *i*PrMgCl as an advanced base instead of lithium hexamethyldisilazane (LHMDS) resulted in dramatic improvements in aza-Claisen rearrangement. This advance is considered responsible for the increased bulkiness of the alkoxide moiety (including magnesium cation and ligands), followed by a resultant conformational change of the transition state. To support this hypothesis, various substrates of aza-Claisen rearrangement were prepared and screened. In addition, a molecular dynamic simulation study was performed to investigate and compare the structural stability of reaction intermediates.

## 1. Introduction

**Figure 1:**
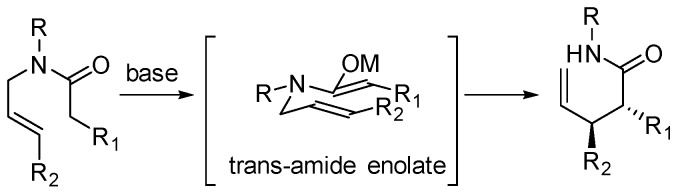
Aza-Claisen rearrangement (ACR) through amide enolate and its stereochemical outcome.

Azacycles, nitrogen containing cyclic molecules, has important biological activity and synthetic utility [1]. Various conversions of azacycle skeletons have contributed to the construction of alkaloid frameworks and the development of important synthetic methodologies (the aza-Cope rearrangement [2], transannulation [3], Diels–Alder cycloaddition [4], and so on [5]). However, their synthetic applications require further development to improve chemical yields, handling, and substrate generality. Aza-Claisen rearrangement (ACR) is one of these methodologies [6]. The [3,3]-sigmatropic rearrangement of nitrogen containing a diene moiety serves as a robust platform to introduce various alkaloid skeletons into natural products or active pharmaceutical ingredients (APIs) [7]. More importantly, with ACR employed, chiral communication and induction of remote stereogenic centers were also reported in a highly selective manner [8,9] (Figure 1). However, this type of rearrangement also requires harsh reaction conditions and at times results in low yields [10].

**Figure 2:**
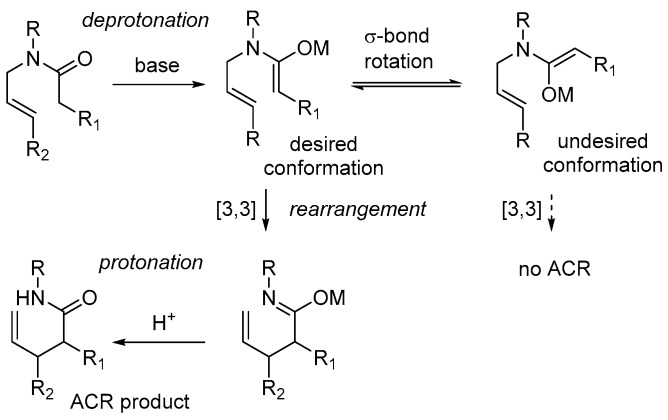
Equilibrium of an amide enolate conformation.

ACR proceeds through deprotonation, [3,3]-sigmatropic rearrangement, and protonation, as shown in Figure 2. For this process, both a strong base for deprotonation of α-hydrogen in an amide group and a high temperature for thermal rearrangement are required [11,12]. However, this reaction condition also permits sigma-bond rotation in amide enolate to hamper generation of the desired conformation. If the undesired conformation exists as a major form, ACR would be impossible (low conversion), and an ensuing side reaction would occur (side product). Because the oxygen–metal bond may play an important role in this enolate conformation, it can be presumed that the cation of a base determines a successful ACR process. Actually, it was shown that lithium hexamethyldisilazane (LHMDS) and *i*PrMgCl gave different results in the ACR of an API synthesis [13].

Inspired by this base-dependent ACR, we tried to apply this methodology to a more general substrate. It was expected that controlling an amide enolate conformation’s equilibrium through various enolate moieties, including metal cations and ligands, would mitigate the undesired side reaction in accelerated ACR processes. Based on this hypothesis, a simple ACR substrate was designed to screen bases.

## 2. Results

**Scheme 1:**
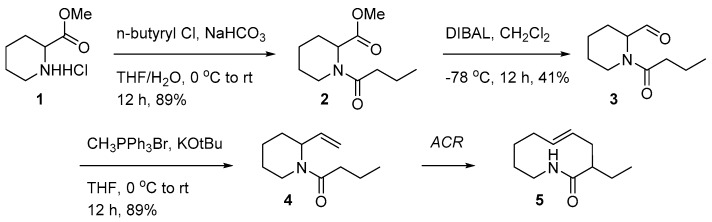
Preparation of substrate for ACR. DIBAL: diisobuylaluminum hydride; THF: tetrahydrofuran.

To prove the efficiency of the cation-dependent ACR, large amounts of substrate were pursued in a short time. In addition, cyclic ACR substrate was favored over acyclic because the ACR of cyclic substrates proceeds with fewer entropic/geometric issues [12]. Commercially available methyl pipecolinate **1** was subjected to the sequential Schotten–Baumann amidation [14] to create amide **2**, which was converted to aldehyde **3** via diisobuylaluminum hydride (DIBAL) reduction. Finally, Wittig methylenation afforded allyl amide **4** an excellent yield, as depicted in Scheme 1.

Various conditions were screened to effectively convert allyl amide **4** to the ring-expanded lactam **5**, as shown in Table 1. As expected, bulky Grignard reagents, such as *i*PrMgCl or *t*BuMgBr, exhibited greater efficiency than LHMDS, the standard base for ACR [15,16] (entries 1, 2, 4). In contrast, *sp^2^*-hybridized carbanion, such as 2-mesitylMgBr, produced a lower yield than LHMDS [17] (entry 3). Relatively small Grignard reagents were also not effective, likely because of the nucleophilic substitution of Grignard reagents. Although sodium and potassium cations are larger than lithium cations, employing NaHMDS or KHMDS as a base also generated worse results than LHMDS (entries 7, 8): in these cases, rather than the ACR adduct **5**, an unidentified side product was obtained soon after the base was added. By contrast, *i*PrMgCl improved the ACR process, but other reaction conditions, such as the solvent and temperature, needed to be further optimized. The screening of representative solvents (benzene, tetrahydrofuran (THF), *n*-decane) is depicted in entries 9–11. The use of benzene improved results slightly; however, polar or extremely nonpolar solvents did not. Lastly, an ACR carried out at room temperature with benzene yielded no reaction (entry 12). Replacing *i*PrMgCl with *i*PrMgBr showed similar results, as shown in entry 13. 

**Scheme 2:**
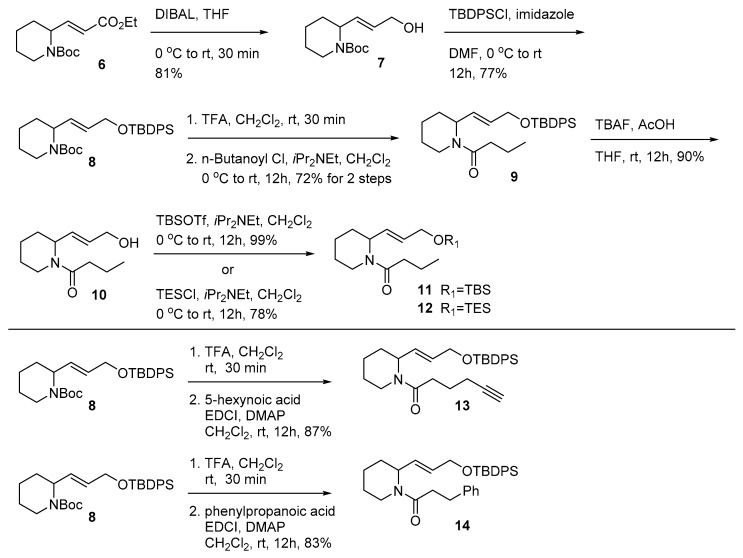
Preparation of various substrates in alkene moiety. TFA: trifluoroacetic acid; TBAF: tetra-*n*-butylammonium fluoride; EDCI: *N*′-(3-dimethylaminopropyl)carbodiimide hydrochloride; DMAP: 4-dimethylaminopyridine; TBS: *tert*-butyldimethylsilyl.

The optimized ACR conditions were applied to various substrates, as shown in Scheme 2. The reduction of known ester **6** [18] provided primary alcohol **7**, which was protected by a *tert-*butyldiphenylsilyl (TBDPS) group. The silyl ether **8** was treated with trifluoroacetic acid (TFA) to generate amine salt, which was transformed uneventfully into butyryl amide **9**. To verify the bulkiness of allyl side chain **9**, TBDPS was converted into *tert*-butyldimethylsilyl (TBS) or triethylsilyl (TES) through tetra-*n*-butylammonium fluoride (TBAF)/AcOH deprotection followed by a corresponding silyl protection. This straightforward synthesis enabled TBS or TES to substitute for ACR substrates **11** and **12** in an excellent yield. Amide substitution was also attempted. After acidic deprotection of the *tert*-butyloxycarbonyl (Boc) group of carbamates **8**, as described above, a concomitant acylation with alkynyl or aromatic functionality was also executed, employing *N*-ethyl-*N′*-(3-dimethylaminopropyl)carbodiimide hydrochloride(EDCI)/4-dimethylaminopyridine (DMAP) amidation conditions. After two uneventful procedures, desired substrates **13** and **14** were produced.

**Scheme 3:**
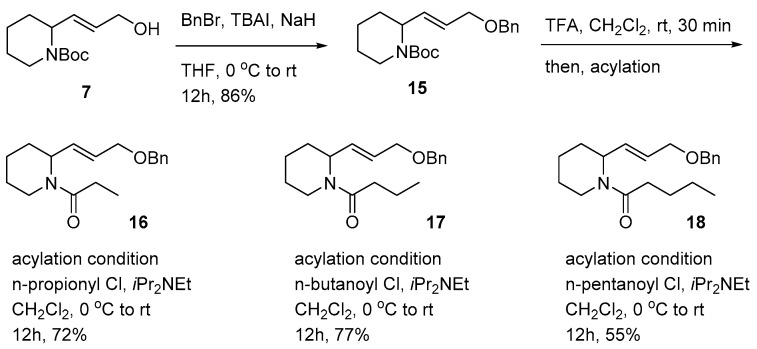
Preparation of various ACR substrates in amide moiety. TBAI: tetra-*n*-butylammonium iodide.

Amide substituent derivatization was also carried out, as shown in Scheme 3. Williamson etherification of the primary alcohol **7**, followed by sequential Boc deprotection/amidation, afforded differently substituted amides **16**–**18** in a straightforward manner. These three derivatives were expected to prove the substituent effect of amide moiety.

**Scheme 4:**
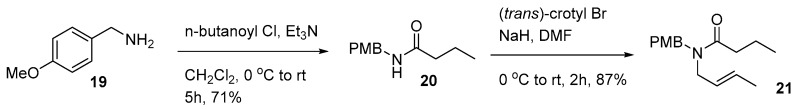
Preparation of acyclic ACR substrates. DMF: tetra-*n*-butylammonium fluoride; PMB: *para*-nethoxyl benzyl.

Finally, acyclic substrate **21** was prepared from *p*-methoxybenzyl amines **19** through an amidation/crotylation sequence. Thus, with the various substrates in hand, *i*PrMgCl mediated ACR (Scheme 4).

**Scheme 5:**
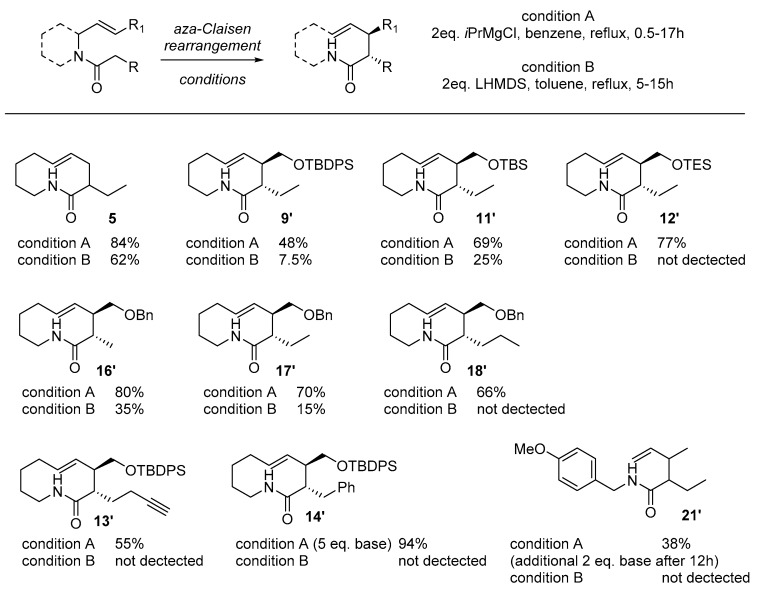
Comparison of ACR conditions using various substrates.

The ACR of various substrates is summarized in Scheme 5. As expected, the employment of *i*PrMgCl/benzene converted each of the substrates into the requisite lactams/amides more efficiently than in conventional conditions (LHMDS/toluene). Of note, superior results occurred in some cases, such as sterically demanded substrates **13** and **14**, as well as labile TES-protected substrate **12**. ACR under the treatment of acyclic amide **21** with *i*PrMgCl also yielded impressive results. Moreover, the LHMDS base did not produce the desired free amide **21’** at all. In fact, a brief comparison of the alkene-substituted ACR substrate showed that the larger the substituent of the allylic side chain, the worse the product yield. Based on this tendency, the increased bulkiness of the side chain could cause the desired ACR process to occur. Similarly, the results from amide analogs **16**–**18** also supported this hypothesis. Consequently, increased repulsion of the allylic side chain with amide enolate is thought to be responsible for the low yield of ACR, but this obstacle can be overcome by employing *i*PrMgCl as a base.

**Figure 3:**
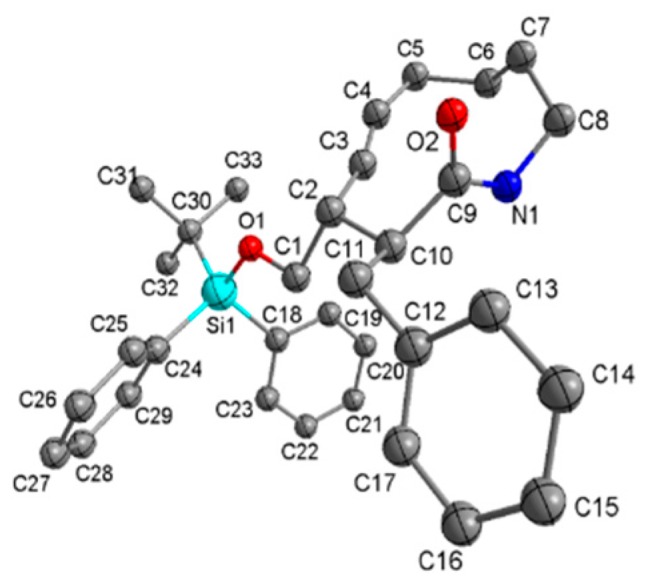
Crystal structure of macrolactam **14’**.

To verify the structure of macrolactams, ACR product **14’** was crystallized. Figure 3 represents the structure of **14’**, which features the 1,2-*anti*-configured side chain and the 10-membered lactam framework. The crystal structure below shows that ACR proceeded as designed.

**Scheme 6:**
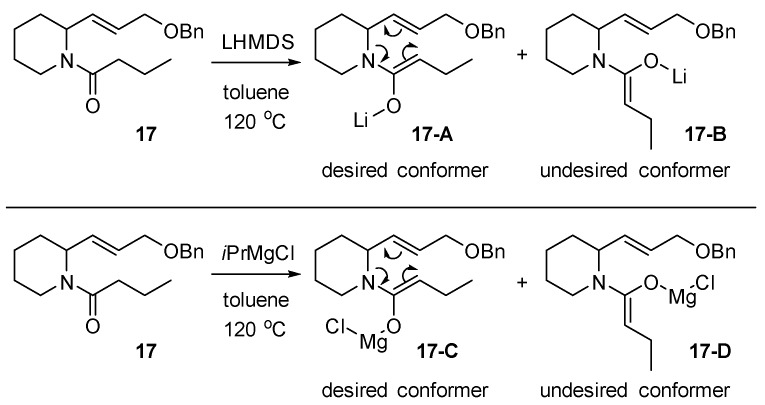
Mechanistic view of cation-dependent ACR.

From a mechanistic point of view, it was hypothesized that reactive conformation would explain the pivotal role of *i*PrMgCl. For example, in the case of amide **17**, a reactive enolate had to be generated and arranged for the desired [3,3]-sigmatropic rearrangement to occur. While desired conformer **17-A** might produce a corresponding ACR product, the undesired conformer **17-B** might lead to no ACR process. The portion of the undesired conformer **17-B** that resulted from the substrate **17** could be lessened when the Grignard base was used instead of LHMDS, as steric repulsion of the alkenyl side chain with Cl-Mg-O complex (in **17-D**) made it more unstable than the Li-O complex did (in **17-B**). This repulsion might give rise to the rapid conversion of the undesired conformer **17-D** to the desired conformer **17-C**, which would result in the ACR process (Scheme 6).

**Figure 4:**
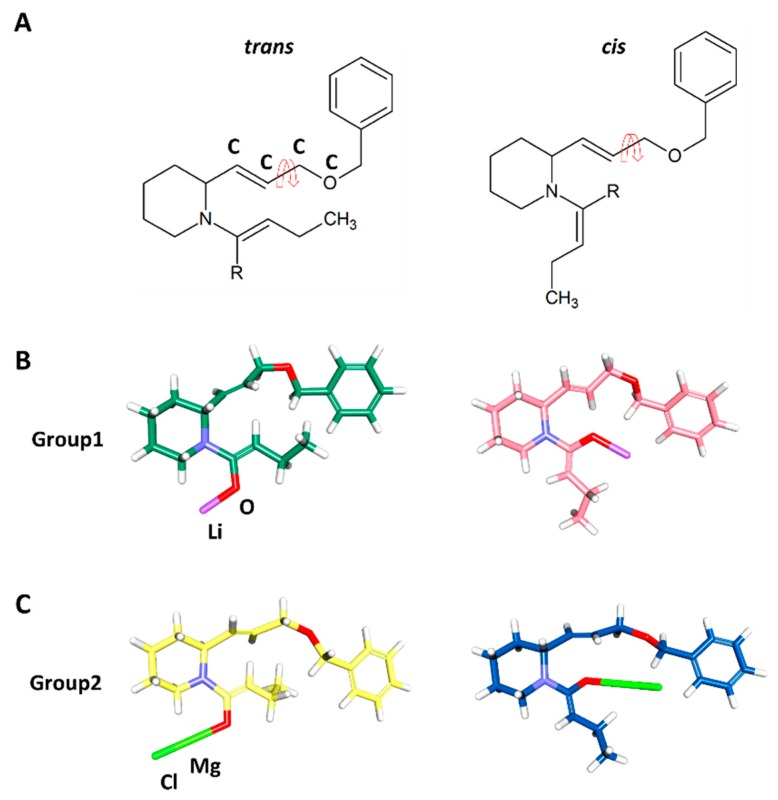
2D and 3D structures of four different conformations of 19 compound. (**A**) Sketch of *trans*, *cis* conformations and C-C-C-O dihedral angle. 3D structure of each compound in (**B**) Group1 and (**C**) Gruop2.

To investigate the structural stability of the reaction intermediates and their conformations (**17-A** through **17-D**), molecular dynamics (MD) simulations were carried out for 10 ns. To monitor the structural stability of each conformation, the C-C-C-O dihedral angles were measured during the simulations (Figure 4).

**Figure 5:**
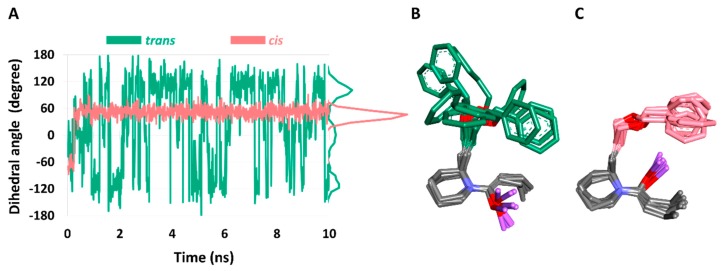
Comparison of structural stability of *trans* and *cis* conformations of Group1 compound. (**A**) Dihedral angle in CCCO of *trans* and *cis* conformations. Cartoon of structure superposition of every nano second frames of cluster obtained from (**B**) *trans* (green) and (**C**) *cis* conformations (pink).

In the case of lithium enolate (Figure 5), MD results clearly showed that the desired Li-conformer **17-A** is very unstable: it did not stay in one state for long (Figure 5A,B). In contrast, the conformation of **17-B** was stable, maintaining the C-C-C-O dihedral angle at 60° (Figure 5A,C).

**Figure 6:**
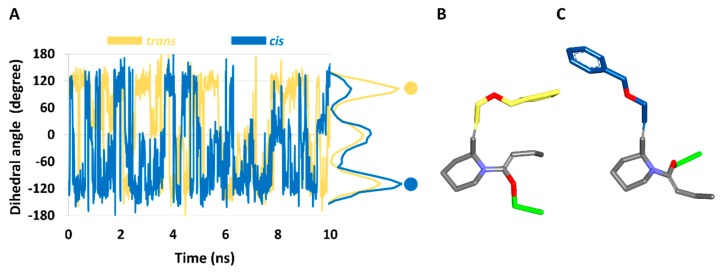
Comparison of structural stability of *trans* and *cis* conformations in Group2 compound. (**A**) Dihedral angle in CCCO of *trans* and *cis* conformations. Cartoon of structure of the most popular clusters obtained from (**B**) *trans* (yellow) and (**C**) *cis* conformations (blue).

However, in the case of magnesium enolate from amide **17**, the structural stability of both conformations showed similar fluctuation patterns (Figure 6A). The most common dihedral angles (i.e., conformation) of **16-C** were 110° (Figure 6A,B), while those of **17-D** were –110° (Figure 6A,C). The instability of undesired conformer **17-D** would explain the rapid conversion of desired conformer **17-C** in the corresponding ACR product.

## 3. Materials and Methods 

### 3.1. General Information

Unless noted otherwise, all starting materials and reagents were obtained from commercial suppliers and were used without further purification. Tetrahydrofuran was distilled from sodium benzophenone ketyl. Dichloromethane was freshly distilled from calcium hydride. All solvents used for routine isolation of products and chromatography were reagent grade. Air- and moisture-sensitive reactions were performed under argon atmosphere. Flash column chromatography was performed using silica gel 60 (230–400 mesh, Merck, Darmstadt, Germany) with the indicated solvents. Thin*-*layer chromatography was performed using 0.25 mm silica gel plates (Merck). ^1^H-NMR data were reported in the order of chemical shift, multiplicity (s, singlet; d, doublet; t, triplet; q, quartet; m, multiplet and/or multiple resonance), number of protons, and coupling constant in hertz (Hz).

#### 3.1.1. Methyl 1-butyrylpiperidine-2-carboxylate (**2**)

To a solution of methyl pipecolinate hydrochloride **1** (3.9 g, 22 mmol) in THF (40 mL), a solution of Na_2_CO_3_ (11 g, 110 mmol) in H_2_O was added at 0 °C. After addition of n-butyryl chloride (2.7 mL, 26 mmol) at 0 °C, the reaction mixture was stirred for 24 h. After filtration of insoluble solids, reaction mixture was extracted EtOAc twice. Organic layers were dried over MgSO_4_, filtered, evaporated, and purified by column chromatography on silica gel (EtOAc:*n*-hexane = 1:1) to afford methyl ester **2** (4.1 g, 89%) as a colorless oil. IR (KBr) ν_max_ 2953, 2869, 1741, 1648, 1426, 1322 cm^−1^; ^1^H-NMR (CDCl_3_, 400 MHz, mixture of rotamers) δ 5.25 (d, 1H, *J* = 5.2 Hz), 4.47–4.40, 3.65–3.61 (m, 1H), 3.57 (s, 3H), 3.09 (dt, 1H, *J* = 2.8, 12.8 Hz), 2.48–2.47, 2.18–2.03 (m, 2H), 2.24 (t, 2H, *J* = 7.6 Hz), 1.59–1.43 (m, 4H), 1.36–1.13 (m, 2H), 0.83 (t, 3H, *J* = 7.6 Hz); ^13^C-NMR (CDCl_3_, 100 MHz, mixture of rotamers) δ 172.7, 172.3, 171.6, 171.1, 55.7, 52.0, 51.7, 51.4, 43.1, 38.9, 35.0, 34.7, 27.0, 26.3, 25.0, 24.3, 20.7, 20.6, 18.2, 13.5; LRMS (FAB) *m*/*z* 214 (M + H^+^); HRMS (FAB) calcd for C_11_H_20_NO_3_ (M + H^+^): 214.1443, found 214.1448.

#### 3.1.2. 1-Butyrylpiperidine-2-carbaldehyde (**3**)

To a solution of methyl ester **2** (850 mg, 4.0 mmol) in CH_2_Cl_2_ (10 mL), DIBAL (1.0 M in toluene, 8.0 mL, 8.0 mmol) was added at –78 °C and stirred for 3 h. Then, 15% sodium potassium tartrate solution (10 mL) was added to reaction mixture and stirred for 12 h at room temperature. Reaction mixture was extracted CH_2_Cl_2_ twice. Organic layers were dried over MgSO_4_, filtered, evaporated, and purified by column chromatography on silica gel (EtOAc:*n*-hexane = 1:1) to afford aldehyde **3** (300 mg, 41%) as a colorless oil. IR (KBr) ν_max_ 2939, 2869, 1731, 1643, 1425 cm^−1^; ^1^H-NMR (CDCl_3_, 400 MHz, mixture of rotamers) δ 9.62 (s), 9.45 (s, 1H), 5.33 (d, *J* = 5.2 Hz), 5.07–5.05 (m, 1H), 4.62–4.58 (m), 4.35–4.33 (m), 3.72–3.64 (m, 2H), 3.08 (dt, 1H, *J* = 2.8, 11.6 Hz), 2.35–2.13 (m, 4H), 1.68–1.53 (m, 5H), 1.43–1.18 (m, 2H), 0.89 (t, 3H, *J* = 7.2 Hz); ^13^C-NMR (CDCl_3_, 100 MHz, mixture of rotamers) δ 201.0, 200.2, 173.1, 62.3, 58.9, 51.9, 51.6, 44.1, 43.3, 35.2, 35.0, 26.5, 25.3, 24.5, 23.2, 20.9, 20.8, 18.5, 13.7; LRMS (FAB) *m*/*z* 184 (M + H^+^); HRMS (FAB) calcd for C_10_H_18_NO_2_ (M + H^+^): 184.1338, found 184.1340.

#### 3.1.3. 1-(2-Vinylpiperidin-1-yl)butan-1-one (**4**)

To a suspension of methyl triphenylphosphonium bromide (850 mg, 2.4 mmol) in THF (5 mL), KOtBu (1.0 M in THF, 2.2 mL, 2.2 mmol) was added at 0 °C and stirred for 30 min. After addition of aldehyde **2** (290 mg, 1.6 mmol) in THF (2 mL), reaction mixture was stirred for 10 min and quenched with addition of H_2_O. Reaction mixture was extracted EtOAc twice. Organic layers were dried over MgSO_4_, filtered, evaporated and purified by column chromatography on silica gel (EtOAc:*n*-hexane = 1:2 to 1:1) to afford allyl amide **4** (220 mg, 77%) as a colorless oil. IR (KBr) ν_max_ 2937, 2867, 1645, 1425, 1244 cm^−1^; ^1^H-NMR (CDCl_3_, 400 MHz, mixture of rotamers) δ 5.71–5.68 (m, 1H), 5.17 (m, 1H), 5.34 (bs), 3.61–3.58 (m, 1H), 5.02–4.98 (m, 1H), 4.48 (bs, 1H), 3.08 (t, *J* = 11.6 Hz), 2.60 (t, 1H, *J* = 11.6 Hz), 2.32–2.20 (m, 2H), 1.79–1.76 (m, 1H), 1.66–1.40 (m, 6H), 1.34 (m, 1H), 0.93–0.89 (m, 3H); ^13^C-NMR (CDCl_3_, 100 MHz) δ 172.4, 171.7, 153.5, 136.5, 116.2, 115.8, 54.4, 51.6, 49.5, 43.3, 41.7, 37.2, 35.5, 35.3, 35.0, 30.0, 28.3, 26.3, 25.2, 19.5, 18.8, 13.9; LRMS (FAB) *m*/*z* 182 (M + H^+^); HRMS (FAB) calcd for C_11_H_20_NO (M + H^+^): 182.1545, found 182.1543.

#### 3.1.4. (*E*)-3-ethyl-3,4,7,8,9,10-hexahydroazecin-2(1*H*)-one (**5**)

*Procedure **A***; To a solution of allyl amide **4** (29 mg, 0.15 mmol) in toluene (1 mL), *i*PrMgCl (2.0 M in THF, 0.15 mL, 0.30 mmol) was added at reflux condition. After stirring at same temperature for 30 min, reaction mixture was cooled down to room temperature and quenched with brine and extracted with EtOAc. Organic layers were dried over MgSO_4_, filtered, evaporated and purified by column chromatography on silica gel (EtOAc:*n*-hexane = 2:1) to afford lactam **5** (22 mg, 78%) as an amorphous solid. 

*Procedure **B***; To a solution of allyl amide **4** (29 mg, 0.15 mmol) in toluene (2 mL), LHMDS (1.0 M in n-hexane, 0.30 mL, 0.30 mmol) was added at reflux condition. After stirring at same temperature for 12 h, reaction mixture was cooled down to room temperature and quenched with brine and extracted with EtOAc. Organic layers were dried over MgSO_4_, filtered, evaporated, and purified by column chromatography on silica gel (EtOAc:*n*-hexane = 2:1) to afford lactam **5** (18 mg, 62%) as an amorphous solid. IR (KBr) ν_max_ 3315, 2925, 2441, 1637, 1550, 1451 cm^−1^; ^1^H-NMR (CD_3_OD, 300 MHz) δ 7.37 (bs, 1H), 5.38–5.16 (m, 2H), 3.49–3.41 (m, 1H), 2.69–2.62 (m, 1H), 2.17–2.10 (m, 1H), 2.07 -1.97 (m, 2H), 1.82–1.74 (m, 3H), 1.67–1.52 (m, 2H), 1.40–1.37 (m, 1H), 1.30–1.19 (m, 2H), 0.77 (t, 3H, *J* = 3.9 Hz); ^13^C-NMR (CDCl_3_, 75 MHz) δ 174.8, 134.5, 127.7, 52.7, 40.2, 37.3, 32.8, 29.7, 29.0, 24.1, 12.4; LRMS (FAB) *m*/*z* 182 (M + H^+^); HRMS (FAB) calcd for C_11_H_20_NO (M + H^+^): 182.1545, found 182.1543.

#### 3.1.5. (*E*)-tert-butyl 2-(3-hydroxyprop-1-enyl)piperidine-1-carboxylate (**7**)

To a solution of unsaturated ester **6** (2.9 g, 10 mmol) in THF (20 mL), DIBAL (1.0 M in toluene, 22 mL, 22 mmol) was added at 0 °C and stirred for 30 min. 15% sodium potassium tartrate solution was added to reaction mixture and stirred for 5 h at room temperature. Reaction mixture was extracted EtOAc twice. Organic layers were dried over MgSO_4_, filtered, evaporated and purified by column chromatography on silica gel (EtOAc:*n*-hexane = 1:1) to afford primary alcohol **7** (2.0 g, 81%) as a colorless oil. IR (KBr) ν_max_ 3443, 2936, 2862, 1690, 1515, 1415, 1367 cm^−1^; ^1^H-NMR (CDCl_3_, 500 MHz, mixture of rotamers) δ 5.64–5.56 (m, 2H), 4.74 (b, 1H) 5.58–5.54 (m, 1H), 4.10–4.09 (m, 2H), 3.88 (d, 2H, *J* = 13.5 Hz), 2.77 (t, 1H, *J* = 12.5 Hz), 2.21 (b, 1H), 1.72–1.58 (m, 2H), 1.54–1. 52 (m, 2H), 1.40 (s, 9H); ^13^C-NMR (CDCl_3_, 100 MHz, mixture of rotamers) δ 171.0, 155.2, 130.4, 129.6, 128.6, 79.3, 62.8, 60.2, 51.4, 50.3, 39.6, 29.0, 28.3, 25.3, 20.8, 19.3, 18.9, 14.0; LRMS (FAB) *m*/*z* 242 (M + H^+^); HRMS (FAB) calcd for C_13_H_24_NO_3_ (M + H^+^): 242.1756, found 242.1752.

#### 3.1.6. (*E*)-tert-butyl 2-(3-(tert-butyldiphenylsilyloxy)prop-1-enyl)piperidine-1-carboxylate (**8**)

To a solution of alcohol **7** (920 mg, 3.8 mmol) in dimethylformamide (DMF, 8 mL), imidazole (390 mg, 5.7 mmol), and TBDPSCl (0.99 mL, 3.8 mmol) were added at 0 °C and stirred for 12 h. After addition of H_2_O, reaction mixture was diluted with EtOAc and washed with H_2_O three times. Organic layers were dried over MgSO_4_, filtered, evaporated, and purified by column chromatography on silica gel (EtOAc:*n*-hexane=1:10 to 1:5) to afford TBDPS ether **8** (1.4 g, 77%) as a colorless oil. IR (KBr) ν_max_ 3431, 2934, 2857, 1692, 1469, 1424 cm^−1^; ^1^H-NMR (CDCl_3_, 300 MHz, mixture of rotamers) δ 7.67–7.65 (m, 4H), 7.42–7.32 (m, 6H), 5.66 (dd, 1H, *J* = 4.8, 15.6 Hz), 5.58–5.54 (m, 1H), 4.76 (s, 1H), 4.20 (dd, 2H, *J* = 1.5, 4.4 Hz), 3.90 (d, 1H, *J* = 12Hz), 2.76 (t, 1H, *J* = 12.7 Hz), 1.67–1.40 (m, 6H), 1.42 (s, 9H), 1.03 (s, 9H); ^13^C-NMR (CDCl_3_, 100 MHz, mixture of rotamers) δ 170.9, 15.2, 135.4, 133.6, 130.0, 129.5, 128.6, 127.5, 79.0, 64.0, 60.2, 29.3, 28.3, 26.7, 25.4, 20.8, 19.4, 19.1, 14.0; LRMS (FAB) *m*/*z* 480 (M + H^+^).

#### 3.1.7. (*E*)-1-(2-(3-(tert-butyldiphenylsilyloxy)prop-1-enyl)piperidin-1-yl)butan-1-one (**9**)

To a solution of TBDPS ether **8** (62 mg, 0.13 mmol) in CH_2_Cl_2_ (2 mL), trifluoroacetic acid (2 mL) was added and stirred for 30 min. After evaporation of reaction mixture, the residue was dissolved in CH_2_Cl_2_ (2 mL) and treated with *i*Pr_2_NEt (0.1 mL) and n-butyryl chloride (0.05 mL) at 0 °C and stirred for 12 h. Reaction mixture was quenched with H_2_O and extracted with CH_2_Cl_2_ twice. Organic layers were dried over MgSO_4_, filtered, evaporated, and purified by column chromatography on silica gel (EtOAc:n-hexane = 1:3 to 1:2) to afford butyryl amide **9** (55 mg, 95%) as a colorless oil. IR (KBr) ν_max_ 2935, 2858, 1732, 1644, 1427, 1238 cm^−1^; ^1^H-NMR (CDCl_3_, 500 MHz, mixture of rotamers) δ 7.68–7.62 (m, 4H), 7.43–7.36 (m, 6H), 5.68 (b, 1H), 5.55 (d, 1H, *J* = 15.5 Hz), 4.52 (b, 1H), 4.23 (s, 1H), 3.70–3.59 (m, 1H), 3.07, 2.60 (m, 1H), 2.31 (t, 2H, *J* = 7.5 Hz), 2.33–2.24 (m, 1H), 1.75 (m, 1H), 1.69–1.35 (m, 7H), 1.06 (s, 9H), 0.96 (t, 3H, *J* = 7.5 Hz); ^13^C-NMR (CDCl_3_, 125MHz) δ 177.3, 135.5, 135.4, 133.5, 130.7, 129.6, 128.2, 127.6, 64.0, 63.7, 53.7, 48.7, 37.2, 35.8, 35.6, 35.1, 30.4, 28.9, 26.7, 26.4, 25.3, 19.5, 19.1, 18.9, 18.2, 13.9, 13.6; LRMS (FAB) *m*/*z* 450 (M + H^+^); HRMS (FAB) calcd for C_28_H_40_NO_7_Si (M + H^+^): 450.2828, found 450.2832.

#### 3.1.8. (*E*)-1-(2-(3-hydroxyprop-1-enyl)piperidin-1-yl)butan-1-one (**10**)

To a solution of n-butyrl amide **9** (370 mg, 0.82 mmol) in THF (10 mL), acetic acid (0.1 mL, 1.6 mmol) and TBAF (1.0 M in THF, 1.2 mL, 1.2 mmol) were added and stirred for 30 min. After addition of aq. NaHCO_3_, reaction mixture was extracted with EtOAc three times. Organic layers were dried over MgSO_4_, filtered, evaporated and purified by column chromatography on silica gel (EtOAc:MeOH = 20:1) to afford primary alcohol **10** (160 mg, 92%) as a colorless oil. IR (KBr) ν_max_ 3398, 2936, 2865, 1620, 1436, 1253 cm^−1^; ^1^H-NMR (CDCl_3_, 400 MHz, mixture of rotamers) δ 5.66–5.58 (m, 2H), 5.31, 4.49 (bs, 1H), 4.43, 3.39 (d, 1H, *J* = 12.8 Hz), 3.09, 2.60 (t, 1H, *J* = 12.4 Hz), 2.26 (t, 2H, *J* = 7.2 Hz), 2.26–2.19 (m, 1H), 1.74 (m, 1H), 1.64–1.54 (m, 7H), 1.34 (m, 1H), 0.90 (t, 3H, *J* = 6.0 Hz); ^13^C-NMR (CDCl_3_, 100 MHz, mixture of rotamers) δ 175.6, 172.4, 171.9, 131.2, 130.9, 129.3, 128.8, 62.9, 62.5, 53.6, 48.8, 41.8, 37.2, 35.5, 35.1, 30.2, 28.7, 26.1, 25.2, 19.4, 18.8, 13.9; LRMS (FAB) *m*/*z* 212 (M + H^+^); HRMS (FAB) calcd for C_12_H_22_NO_2_ (M + H^+^): 212.1651, found 212.1644.

#### 3.1.9. (*E*)-1-(2-(3-(tert-butyldimethylsilyloxy)prop-1-enyl)piperidin-1-yl)butan-1-one (**11**)

To a solution of alcohol **10** (72 mg, 0.34 mmol) in CH_2_Cl_2_ (2 mL), *i*Pr_2_NEt (0.2 mL) and TBSOTf (0.05 mL) were added at 0 °C and stirred for 12 h. After addition of H_2_O, reaction mixture was extracted with CH_2_Cl_2_. Organic layers were dried over MgSO_4_, filtered, evaporated, and purified by column chromatography on silica gel (EtOAc:*n*-hexane = 1:3 to 1:2) to afford TBS ether **11** (110 mg, 99%) as a colorless oil. IR (KBr) ν_max_ 2934, 2857, 1645, 1539, 1424 cm^−1^; ^1^H-NMR (CDCl_3_, 400 MHz, mixture of rotamers) δ 5.59–5.49 (m, 2H), 5.35 (bs), 3.59–3.57 (m, 1H), 4.49–4.45 (m, 1H), 4.13 (s, 2H), 3.09, 259 (bs, 1H), 2.26 (t, 1H, *J* = 5.6 Hz), 2.20 (bs, 1H), 1.73 (m, 1H), 1.64–1.50 (m, 6H), 1.34 (b, 1H), 0.90 (t, 3H, *J* = 4.6 Hz), 0.85 (s, 9H), 0.00 (s, 6H); ^13^C-NMR (CDCl_3_, 100 MHz, mixture of rotamers) δ 172.2, 171.5, 131.1, 128.0, 127.8, 63.3, 62.9, 53.6, 48.6, 41.7, 37.2, 35.5, 35.1, 30.3, 28.8, 26.3, 25.8, 25.3, 19.5, 18.8, 18.2, 13.9; LRMS (FAB) *m*/*z* 326 (M + H^+^); HRMS (FAB) calcd for C_18_H_36_NO_2_Si (M + H^+^): 326.2515, found 326.2520.

#### 3.1.10. (*E*)-1-(2-(3-(triethylsilyloxy)prop-1-enyl)piperidin-1-yl)butan-1-one (**12**)

To a solution of alcohol **10** (100 mg, 0.47 mmol) in CH_2_Cl_2_ (2 mL), *i*Pr_2_NEt (0.17 mL, 0.95 mmol) and TESCl (1.0 M in THF, 0.62 mL, 0.62 mmol were added at 0 °C and stirred for 2 h. After addition of H_2_O, reaction mixture was extracted with CH_2_Cl_2_. Organic layers were dried over MgSO_4_, filtered, evaporated and purified by column chromatography on silica gel (EtOAc:*n*-hexane = 1:3 to 1:2) to afford TES ether **12** (120 mg, 78%) as a colorless oil. IR (KBr) ν_max_ 2955, 1645, 1537, 1458, 1118 cm^−1^; ^1^H-NMR (CDCl_3_, 300 MHz, mixture of rotamers) δ 5.57–5.36 (m, 2H), 4.50 (b, 1H), 4.13 (s, 2H), 3.57–3.47 (m), 3.41–3.37 (m, 1H), 3.10 (t, *J* = 12.0 Hz), 2.60 (t, 1H, *J* = 12.0 Hz), 2.27 (t, 2H, *J* = 7.5 Hz), 1.74–1.35 (m, 7H), 0.92 (t, 9H, *J* = 8.1 Hz), 0.57 (q, 6H, *J* = 8.1 Hz); LRMS (FAB) *m*/*z* 326 (M + H^+^); HRMS (FAB) calcd for C_18_H_36_NO_2_Si (M + H^+^): 326.2515, found 326.2519.

#### 3.1.11. (*E*)-1-(2-(3-(tert-butyldiphenylsilyloxy)prop-1-enyl)piperidin-1-yl)hex-5-yn-1-one (**13**)

To a solution of TBDPS ether **8** (72 mg, 0.15 mmol) in CH_2_Cl_2_ (1 mL), trifluoroacetic acid (1 mL) was added and stirred for 30 min. After evaporation of reaction mixture, the residue was dissolved in CH_2_Cl_2_ (2 mL) and treated with EDCI (57 mg, 0.3 mmol), DMAP (37 mg, 0.3 mmol) and 5-hexynoic acid (33 μL, 0.3 mmol) at 0 °C and stirred for 12 h. Reaction mixture was quenched with H_2_O and extracted with CH_2_Cl_2_ twice. Organic layers were dried over MgSO_4_, filtered, evaporated, and purified by column chromatography on silica gel (EtOAc:*n*-hexane = 1:3 to 1:2) to afford 5-hexynyl amide **13** (62 mg, 87%) as a colorless oil. IR (KBr) ν_max_ 3300, 2935, 2857, 1642, 1428, 1256 cm^−1^; ^1^H-NMR (CDCl_3_, 400 MHz, mixture of rotamers) δ 7.66–7.60 (m, 4H), 7.42–7.24 (m, 6H), 5.71–5.63 (m, 1H), 5.57–5.50 (m, 1H), 4.53–4.45 (m, 1H), 4.11 (d, 2H, *J* = 7.2 Hz), 3.69–3.60 (m, 1H), 3.08–3.02 (m, 1H), 2.47–2.44 (m, 2H), 2.32 (b, 2H), 1.94–1.51 (m, 9H), 1.12 (s, 9H); ^13^C-NMR (CDCl_3_, 100 MHz, mixture of rotamers) δ 135.6, 135.4, 133.5, 130.8, 129.6, 127.6, 83.8, 68.8, 63.8, 31.9, 31.4, 30.4, 28.9, 26.7, 26.3, 23.9, 19.5, 19.2 18.0; LRMS (FAB) *m*/*z* 474 (M + H^+^); HRMS (FAB) calcd for C_30_H_40_NO_7_Si(M + H^+^): 474.2828, found 474.2816.

#### 3.1.12. (*E*)-1-(2-(3-(tert-butyldiphenylsilyloxy)prop-1-enyl)piperidin-1-yl)hex-5-yn-1-one (**14**)

To a solution of TBDPS ether **8** (1.0 g, 2.1 mmol) in CH_2_Cl_2_ (10 mL), trifluoroacetic acid (5 mL) was added and stirred for 1 h. After addition of aq. Na_2_CO_3_, reaction mixture was extracted with CH_2_Cl_2_. Organic layers were dried over MgSO_4_, filtered, evaporated, and dissolved in CH_2_Cl_2_ (20 mL). To this solution, 3-phenylpropionic acid (0.5 g, 3.6 mmol), EDCI (0.7 g, 3.6 mmol) and DMAP (0.9 g, 7.2 mmol) were added and stirred for 12 h. Reaction mixture was quenched with aq. NH_4_Cl and extracted with CH_2_Cl_2_. Organic layers were dried over MgSO_4_, filtered, evaporated, and purified by column chromatography on silica gel (EtOAc:*n*-hexane = 1:5) to afford amide **14** (0.9 g, 83% for 2 steps) as a pale yellow oil. ^1^H-NMR (CDCl_3_, 300 MHz, mixture of rotamers) δ 7.77–7.63 (m, 4H), 7.49–7.35(m, 6H), 7.33–7.18 (m, 5H), 5.74–5.43 (m, 2H), 4.50 (s, 1H), 4.25 (s, 2H), 3.46 (m, 1H), 3.00 (t, 2H, *J* = 7.8, 15.9 Hz), 2.67 (t, 2H, *J* = 7.5, 15.6 Hz), 1.48 (m, 6H), 1.07 (m, 10H); ^13^C-NMR (CDCl_3_, 125 MHz, mixture of rotamers) δ 71.6, 170.9, 141.3, 135.5, 134.7, 134.6, 133.6, 130.9, 129.7(2), 129.6, 128.5, 128.4, 128.0, 127.8, 127.6, 127.4(2) 126.1, 77.3, 77.2, 77.0, 76.8, 64.1, 63.7, 53.9, 49.1, 41.8, 37.6, 34.9, 31.7, 30.3, 26.8, 26.2, 25.9, 25.8, 25.2, 19.5, 19.2, 18.6, 18.5. LRMS (EI) *m*/*z* 512 (M + H^+^); HRMS (EI) calcd for C_33_H_41_O_2_NSi (M^+^): 511.2907, found 511.2903.

#### 3.1.13. (*E*)-tert-butyl 2-(3-(benzyloxy)prop-1-enyl)piperidine-1-carboxylate (**15**)

To a solution of alcohol **7** (690 mg, 3.2 mmol) in THF (15 mL), TBAI (120 mg, 0.32 mmol), NaH (60% mineral oil, 150 mg, 3.7 mmol) and benzyl bromide (0.42 mL, 3.5 mmol) were added at 0 °C and stirred for 12 h at room temperature. After addition of H_2_O, reaction mixture was extracted with EtOAc twice. Organic layers were dried over MgSO_4_, filtered, evaporated and purified by column chromatography on silica gel (EtOAc:*n*-hexane = 1:10 to 1:5) to afford benzyl ether **15** (920 mg, 86%) as a colorless oil. IR (KBr) ν_max_ 2935, 2857, 1691, 1453, 1409, 1365 cm^−1^; ^1^H-NMR (CDCl_3_, 400 MHz, mixture of rotamers) δ 7.31–7.22 (m, 5H), 5.68–5.55 (m, 2H), 4.80 (s, 1H), 4.48 (d, 2H, *J* = 4.8 Hz), 4.01–4.00 (m, 2H), 3.92 (d,1H, *J* = 13.2 Hz), 2.80 (t, 1H, *J* = 11.6 Hz), 1.72–1.62 (m, 2H), 1.60–1.50 (m, 2H), 1.47–1.31 (m, 2H), 1.43 (s, 9H); ^13^C-NMR (CDCl_3_, 100 MHz, mixture of rotamers) δ 155.0, 138.1, 131.8, 128.2, 127.5, 127.4, 79.1, 72.3, 71.7, 70.2, 66.1, 39.6, 28.9, 28.3, 25.3, 19.4; LRMS (FAB) *m*/*z* 332 (M + H^+^); HRMS (FAB) calcd for C_20_H_30_NO_3_ (M + H^+^): 332.2226, found 332.2226.

#### 3.1.14. (*E*)-1-(2-(3-(benzyloxy)prop-1-enyl)piperidin-1-yl)propan-1-one (**16**)

Benzyl ether **15** (120 mg, 0.36 mmol) was converted to propyl amide **16** (75 mg, 72%) using same procedure as described in Section 3.1.7. IR (KBr) ν_max_ 2936, 2857, 1644, 1538, 1426, 1251 cm^−1^; ^1^H-NMR (CDCl_3_, 400 MHz, mixture of rotamers) δ 7.33–7.11 (m, 5H), 5.70–5.55 (m, 2H), 5.39 (bs), 3.60 (d, 1H, *J* = 11.8 Hz), 4.48 (s, 2H), 4.48 (bs, 1H), 3.97 (d, 2H, *J* = 5.8 Hz), 3.09 (t, *J* = 12.3 Hz), 2.62 (t, 1H, *J* = 12.3 Hz), 2.34–2.27 (m, 2H), 1.79–1.76 (m, 1H), 1.63–1.52 (m, 4H), 1.27 (m, 1H), 1.09 (t, 3H, *J* = 6.0 Hz); ^13^C-NMR (CDCl_3_, 100 MHz, mixture of rotamers) δ 173.0, 172.4, 138.1, 131.4, 130.9, 128.3, 128.0, 127.6, 126.9, 72.2, 72.0, 70.3, 69.9, 53.5, 48.8, 41.6, 37.3, 30.1, 28.5, 26.7, 26.2, 25.2, 19.6, 9.4, 9.0; LRMS (FAB) *m*/*z* 288 (M + H^+^); HRMS (FAB) calcd for C_18_H_26_NO_2_ (M + H^+^): 288.1964, found 288.1956.

#### 3.1.15. (*E*)-1-(2-(3-(benzyloxy)prop-1-enyl)piperidin-1-yl)butan-1-one (**17**)

Benzyl ether **15** (140 mg, 0.42 mmol) was converted to butyl amide **17** (97 mg, 77%) using same procedure as described in Section 3.1.7. IR (KBr) ν_max_ 2935, 2858, 1641, 1425, 1362 cm^−1^; ^1^H-NMR (CDCl_3_, 400 MHz, mixture of rotamers) δ 7.33–7.23 (m, 5H), 5.70–5.60 (m, 2H), 5.39 (bs), 3.60 (d, 1H, *J* = 9.0 Hz), 4.48 (m, 3H), 4.01 (d, 2H, *J* = 3.2 Hz), 3.09 (t, *J* = 12.3 Hz), 2.58 (t, 1H, *J* = 12.3 Hz), 2.31–2.21 (m, 2H), 1.79–1.76 (m, 1H), 1.67–1.58 (m, 6H), 1.36 (m, 1H), 0.93 (t, 3H, *J* = 7.2 Hz); ^13^C-NMR (CDCl_3_, 100 MHz, mixture of rotamers) δ 176.6, 172.3, 171.8, 138.1, 137.9, 131.4, 130.9, 128.3, 127.7, 127.6, 72.2, 71.9, 70.3, 69.9, 53.6, 48.7, 41.8, 37.2, 35.8, 35.5, 35.1, 30.2, 28.6, 26.2, 25.2, 19.6, 19.4, 18.8, 18.6, 18.2, 13.9, 13.5; LRMS (FAB) *m*/*z* 302 (M + H^+^); HRMS (FAB) calcd for C_19_H_28_NO_2_ (M + H^+^): 302.2120, found 302.2120.

#### 3.1.16. (*E*)-1-(2-(3-(benzyloxy)prop-1-enyl)piperidin-1-yl)pentan-1-one (**18**)

Benzyl ether **15** (190 mg, 0.57 mmol) was converted to pentyl amide **18** (99 mg, 55%) using same procedure as described in Section 3.1.7. IR (KBr) ν_max_ 2933, 2858, 1642, 1424, 1263 cm^−1^; ^1^H-NMR (CD_3_OD, 400 MHz, mixture of rotamers) δ 7.32–7.23 (m, 5H), 5.80–5.56 (m, 2H), 5.29 (bs), 4.67 (bs, 1H), 4.49 (s, 2H), 4.40 (d, *J* = 12.3 Hz), 3.73 (d, 1H, *J* = 12.4 Hz), 4.01 (d, 2H, *J* = 3.2 Hz), 3.15 (t, *J* = 12.9 Hz), 2.58 (t, 1H, *J* = 12.5 Hz), 2.45–2.25 (m, 2H), 1.82 (b, 1H), 1.68–1.53 (m, 6H), 1.39–1.20 (m, 3H), 0.93–0.86 (m, 3H); ^13^C-NMR (CDCl_3_, 100 MHz, mixture of rotamers) δ 176.4, 172.4, 1719, 138.1, 131.4, 131.0, 128.3, 128.0, 127.5, 72.0, 70.3, 69.9, 53.7, 48.7, 41.8, 37.3, 33.6, 33.3, 32.9, 30.2, 28.6, 27.5, 26.8, 26.3, 25.2, 22.5, 22.1, 19.6, 14.0, 13.6; LRMS (FAB) *m*/*z* 316 (M + H^+^); HRMS (FAB) calcd for C_20_H_30_NO_2_ (M + H^+^): 316.2277, found 316.2278.

#### 3.1.17. *N*-(4-methoxybenzyl)butyramide (**20**)

To a solution of 4-methoxybenzylamine **19** (300 mg, 2.2 mmol) in CH_2_Cl_2_ (10 mL), Et_3_N (0.4 mL, 2.8 mmol) and n-butyryl chloride (0.25 mL, 2.4 mmol) were at 0 °C and stirred for 5 h at room temperature. Reaction mixture was quenched with H_2_O and extracted with CH_2_Cl_2_ twice. Organic layers were dried over MgSO_4_, filtered, evaporated, and purified by column chromatography on silica gel (EtOAc:*n*-hexane = 1:1) to afford *para*-nethoxyl benzyl (PMB) amide **20** (320 mg, 71%) as a white amorphous solid. IR (KBr) ν_max_ 3290, 2959, 1632, 1554, 1513 cm^−1^; ^1^H-NMR (CDCl_3_, 400 MHz) δ 7.17 (d, 2H, *J* = 8.6 Hz), 6.83 (d, 2H, *J* = 8.6 Hz), 4.85 (s, 1H), 4.26 (s, 2H), 3.72 (s, 3H), 2.17 (t, 2H, *J* = 7.2 Hz), 1.67–1.58 (m, 2H), 0.92 (t, 3H, *J* = 7.3Hz); ^13^C-NMR (CDCl_3_, 100 MHz) δ 176.4, 161.0, 132.8, 130.6, 115.6, 56.4, 44.4, 39.7, 21.1, 14.8; LRMS (FAB) *m*/*z* 208 (M + H^+^); HRMS (FAB) calcd for C_12_H_18_NO_2_ (M + H^+^): 208.1338, found 208.1334.

#### 3.1.18. *N*-(but-2-enyl)-*N*-(4-methoxybenzyl)butyramide (**21**)

To a solution of PMB amide **20** (115 mg, 0.55 mmol) in DMF (3 mL), NaH (60% mineral oil, 26 mg, 0.66 mmol) and trans-crotyl bromide (0.10 mL, 0.83 mmol) were added at 0 °C and stirred for 2 h at room temperature. After dilution with EtOAc and reaction mixture was washed with H_2_O 3 times. Organic layers were dried over MgSO_4_, filtered, evaporated, and purified by column chromatography on silica gel (EtOAc:*n*-hexane = 1:3) to afford crotyl amide **21** (128 mg, 87%) as a colorless oil. IR (KBr) ν_max_ 2962, 1645, 1513, 1459, 1248 cm^−1^; ^1^H-NMR (CDCl_3_, 400 MHz, mixtures of rotamers) δ 7.14–7.12 (m, 2H), 6.85–6.78 (m, 2H), 5.60–5.45 (m, 1H), 5.44–5.27 (m, 1H), 4.46 (s), 4.39 (s, 2H), 3.99 (d, *J* = 6.8 Hz), 3.79 (d, 1H, *J* = 6.8 Hz), 3.87 (d, *J* = 6.0 Hz), 3.68 (d, 1H, *J* = 6.0 Hz), 3. 76 (s), 3.74 (s, 3H), 2.32–2.27 (m, 2H), 1.71–1.50 (m, 5H), 0.96–0.84 (m, 3H); ^13^C-NMR (CDCl_3_, 100 MHz, mixtures of rotamers) δ 172.9, 172.8, 158.8, 158.7, 130.0, 129.8, 129.4, 128.8, 128.7, 128.1, 127.7, 127.5, 127.4, 127.3, 125.9, 125.8, 125.5, 114.1, 113.7, 55.1, 49.4, 49.1, 48.1, 47.3, 47.0, 46.7, 43.4, 41.2, 35.1, 34.9, 18.7, 17.6, 17.5, 13.9, 12.8; LRMS (FAB) *m*/*z* 262 (M + H^+^); HRMS (FAB) calcd for C_16_H_24_NO_2_ (M + H^+^): 262.1807, found 262.1800.

#### 3.1.19. (Rac-3*S*,4*R*,*E*)-4-((tert-butyldiphenylsilyloxy)methyl)-3-ethyl-3,4,7,8,9,10-hexahydroazecin-2(1*H*)-one (**9’**)

TBDPS ether **9** (27 mg, 0.060 mmol) was converted to macrolactam **9’** as an amorphous solid using *Procedure A* (13 mg, 48%) or *Procedure B* (2 mg, 7.5%). IR (KBr) ν_max_ 3300, 2929, 1645, 1541, 1458 cm^−1^; ^1^H-NMR (CDCl_3_, 300 MHz) δ 7.58–7.56 (m, 4H), 7.36–7.29 (m, 6H), 5.49–5.36 (m, 2H), 4.89 (d, 1H, *J* = 9.0 Hz), 3.71–3.56 (m, 3H), 2.77 (dd, 1H, *J* = 6.9, 12.9 Hz), 2.13 (m, 2H), 1.97 (dt, 2H, *J* = 7.2, 11.6 Hz), 1.89–1.13 (m, 7H), 1.00 (s, 3H), 0.76 (t, 3H, *J* = 7.2 Hz); ^13^C-NMR (CDCl_3_, 75MHz) δ 174.8, 135.6, 133.6, 133.2, 130.4, 129.7, 127.6, 63.7, 53.5, 49.0, 40.3, 33.1, 29.8, 26.9, 26.8, 21.4, 19.4, 12.8; LRMS (FAB) *m*/*z* 450 (M + H^+^); HRMS (FAB) calcd for C_28_H_40_NO_7_Si (M + H^+^): 450.2828, found 450.2832.

#### 3.1.20. (Rac-3*S*,4*R*,*E*)-4-((tert-buyldimethylsilyloxy)methyl)-3-ethyl-3,4,7,8,9,10-hexahydroazecin-2(1*H*)-one (**11’**)

TBS ether **11** (48 mg, 0.15 mmol) was converted to macrolactam **11’** as an amorphous solid using *Procedure A* (33 mg, 69%) or *Procedure B* (12 mg, 25%). IR (KBr) ν_max_ 3294, 2928, 2857, 1644, 1550 cm^−1^; ^1^H-NMR (CDCl_3_, 400 MHz) δ 5.43–5.36 (m, 2H), 4.97 (d, 1H, *J* = 5.2 Hz), 3.71–3.62 (m, 3H), 2.80 (dd, 1H, *J* = 6.0, 10.8 Hz), 2.21–2.13 (m, 2H), 1.97 (dt, 1H, *J* = 3.1, 6.5 Hz), 1.92–1.86 (m, 1H), 1.81–1.76 (m, 1H), 1.71–1.62 (m, 2H), 1.49–1.42 (m, 2H), 1.24–1.17 (m, 1H), 0.87 (s, 9H), 0.84 (t, 3H, *J* = 5.8 Hz), 0.01 (s, 3H), 0.01 (s, 3H); ^13^C-NMR (CD_3_OD, 100 MHz) δ 178.4, 134.3, 133.3, 65.4, 54.8, 51.6, 42.0, 34.4, 31.4, 27.2, 23.1, 20.0, 13.9, −4.3, −4.5; LRMS (FAB) *m*/*z* 326 (M + H^+^); HRMS (FAB) calcd for C_18_H_36_NO_2_Si (M + H^+^): 326.2515, found 326.2514.

#### 3.1.21. (Rac-3*S*,4*R*,*E*)-4-((triethylsilyloxy)methyl)-3-ethyl-3,4,7,8,9,10-hexahydroazecin-2(1*H*)-one (**12’**)

TES ether **12** (40 mg, 0.12 mmol) was converted to macrolactam **12’** as an amorphous solid using *Procedure A* (31 mg, 69%). IR (KBr) ν_max_ 3299, 2926, 1646, 1553, 1453, 137 cm^−1^; ^1^H-NMR (CDCl_3_, 400 MHz) δ 5.45–5.35 (m, 2H), 5.00 (d, 1H, *J* = 8.3 Hz), 3.71–3.60 (m, 3H), 2.80 (dd, 1H, *J* = 7.6, 13.7 Hz), 2.21–2.00 (m, 2H), 1.96 (dt, 1H, *J* = 4.0, 10.2 Hz), 1.92–1.87 (m, 1H), 1.81–1.67 (m, 3H), 1.50–1.42 (m, 2H), 1.25–1.16 (m, 1H), 0.92 (t, 9H, *J* = 8.0 Hz), 0.84 (t, 3H, *J* = 7.2 Hz), 0.56 (q, 6H, *J* = 8.0 Hz); ^13^C-NMR (CDCl_3_, 100 MHz) δ 174.9, 133.0, 130.5, 77.2, 62.5, 53.1, 49.0, 40.3, 33.1, 29.7, 21.4, 12.8, 6.7, 4.3; LRMS (FAB) *m*/*z* 326 (M + H^+^); HRMS (FAB) calcd for C_18_H_36_NO_2_Si (M + H^+^): 326.2515, found 326.2519.

#### 3.1.22. (Rac-3*S*,4*R*,*E*)- 3-(but-3-ynyl)-4-((tert-butyldiphenylsilyloxy)methyl)-3,4,7,8,9,10-hexahydroazecin-2(1*H*)-one (**13’**)

Hexynyl amide **13** (22 mg, 46 μmol) was converted to macrolactam **13’** as an amorphous solid using *Procedure A* (12 mg, 55%). IR (KBr) ν_max_ 3303, 2928, 2857, 1645, 1541, 1430 cm^−1^; ^1^H-NMR (CDCl_3_, 300 MHz,) δ 7.67–7.63 (m, 4H), 7.44–7.33 (m, 6H), 5.51 (dd, 1H, *J* = 8.7, 15.6 Hz), 5.46–5.36 (m, 1H), 5.01 (d, 1H, *J* = 9.6 Hz), 3.71 (d, 2H, *J* =2.7 Hz), 3.67 (m, 1H), 2.81 (dd, 1H, *J* = 7.5, 13.2 Hz), 2.44 (dd, 1H, *J* = 3.0, 10,8 Hz), 2.29–2.15 (m, 3H), 2.10–1.47 (m, 8H), 1.23 (m, 1H), 1.05 (s, 9H); ^13^C-NMR (CDCl_3_, 75 MHz) δ 174.1, 135.7, 135.6, 133.5, 133.4, 130.0, 129.7, 129.6, 127.7, 84.0, 68.6, 63.4, 50.0, 48.9, 40.3, 33.1, 27.0, 26.9, 19.3, 17.0; LRMS (FAB) *m*/*z* 474 (M + H^+^); HRMS (FAB) calcd for C_30_H_40_NO_7_ Si (M + H^+^): 474.2828, found 474.2832.

#### 3.1.23. (Rac-3*S*,4*R*,*E*)- 3-benzyl-4-((tert-butyldiphenylsilyloxy)methyl)-3,4,7,8,9,10-hexahydroazecin-2(1*H*)-one (**14’**)

Phenylethyl amide **14** (1.0 g, 1.9 mmol) was converted to macrolactam **14’** as an amorphous solid using *Procedure A* (0.94 g, 94%). ^1^H-NMR (CDCl_3_, 500 MHz) δ 7.62–7.59 (m, 4H), 7.37–7.25 (m, 6H), 7.14–7.02 (m, 5H), 5.46–5.36 (m, 2H), 4.82 (d, 1H, *J* = 8.0 Hz), 3.84–3.75 (m, 2H), 3.51 (ddd, 1H, *J* = 9.0, 12.5, 17.0 Hz), 2.93 (dd, 1H, *J* = 12.0, 14.0 Hz), 2.61 (dd, 2H, *J* = 2.5, 13.5Hz), 2.57 (dd, 1H, *J* = 11.5, 19Hz), 2.39 (ddd, 1H, *J* = 2.5, 10.5, 13Hz), 2.24 (dd, 1H, *J* = 8.5, 18.5, Hz), 2.14 (dd, 1H, *J* = 3.5, 12.5, Hz) 1.83–1.71 (m, 2H), 1.63 (d, 1H, *J* = 7.0 Hz), 1.40 (dd, 1H, *J* = 11.5, 23.0 Hz), 1.06–0.99 (m, 9H); ^13^C-NMR (CDCl_3_, 125MHz) δ173.9, 141.0, 140.7, 135.7, 133.5, 129.9, 128.9, 128.5, 127.7, 127.0, 125.9, 65.2, 63.6, 53.8, 49.0, 40.3, 34.3, 33.1, 29.8, 27.0, 19.4; LRMS (EI) *m*/*z* 512 (M + H^+^); HR-MS (EI) calcd for C_33_H_41_O_2_NSi (M^+^): 511.2907, found 511.2908.

#### 3.1.24. (Rac-3*S*,4*R*,*E*)- 4-(benzyloxymethyl)-3-methyl-3,4,7,8,9,10-hexahydroazecin-2(1*H*)-one (**16’**)

Propyl amide **16** (20 mg, 70 μmol) was converted to macrolactam **16’** as an amorphous solid using *Procedure A* (16 mg, 80%) or *Procedure B* (7 mg, 35%). IR (KBr) ν_max_ 3312, 2924, 1644, 1550, 1452, 1367 cm^−1^; ^1^H-NMR (CD_3_OD, 300 MHz) δ 7.33–7.24 (m, 5H), 5.52–5.31 (m, 2H), 4.48 (q, 2H, *J* = 12.0 Hz), 3.54 (d, 2H, *J* = 4.5 Hz), 3.57–3.47 (m, 1H), 2.73 (dd, 1H, *J* = 6.9, 13.5 Hz), 2.37–2.12 (m, 3H), 1.93–1.72 (m, 3H), 1.51–1.27 (m, 2H), 1.05 (d, 3H, *J* = 6.3 Hz); ^13^C-NMR (CDCl_3_, 100 MHz) δ 175.6, 138.2, 133.8, 130.3, 128.3, 127.6, 127.5, 126.9, 73.2, 69.7, 48.5, 45.0, 40.1, 33.0, 29.6, 13.6; LRMS (FAB) *m*/*z* 288 (M + H^+^); HRMS (FAB) calcd for C_18_H_26_NO_2_ (M + H^+^): 288.1964, found 288.1956.

#### 3.1.25. (Rac-3*S*,4*R*,*E*)-4-(benzyloxymethyl)-3-ethyl-3,4,7,8,9,10-hexahydroazecin-2(1*H*)-one (**17’**)

Butyryl amide **17** (20 mg, 66 μmol) was converted to macrolactam **17’** as an amorphous solid using *Procedure A* (14 mg, 70%) or *Procedure B* (3 mg, 15%). IR (KBr) ν_max_ 2926, 2856, 2421, 1637, 1457 cm^–1^; ^1^H-NMR (CD_3_OD, 300 MHz) δ 7.24–7.15 (m, 5H), 5.41–5.22 (m, 2H), 4.38 (q, 2H, *J* = 12.0 Hz), 3.44 (d, 2H, *J* = 4.0 Hz), 3.45–3.44 (m, 1H), 2.66 (m, 1H), 2.16–2.02 (m, 3H), 1.78–1.18 (m, 7H), 0.73 (t, 3H, *J* = 7.2 Hz); ^13^C-NMR (CD_3_OD, 75 MHz) δ 178.2, 140.5, 134.5, 133.3, 130.1, 129.7, 129.4, 74.9, 72.3, 55.0, 42.0, 33.9, 31.4, 23.1, 21.4, 14.9, 13.8.; LRMS (FAB) *m*/*z* 302 (M + H^+^); HRMS (FAB) calcd for C_19_H_28_NO_2_ (M + H^+^): 302.2120, found 302.2121. 

#### 3.1.26. (Rac-3*S*,4*R*,*E*)- 4-(benzyloxymethyl)-3-propyl-3,4,7,8,9,10-hexahydroazecin-2(1*H*)-one (**18’**)

Pentyryl amide **18** (44 mg, 0.14 mmol) was converted to macrolactam **18’** as an amorphous solid using *Procedure A* (29 mg, 66%). IR (KBr) ν_max_ 3304, 2927, 2858, 1645, 1543, 1453 cm^−1^; ^1^H-NMR (CD_3_OD, 300 MHz) δ 7.23–7.15 (m, 5H), 5.42–5.22 (m, 2H), 4.43 (d, 1H, *J* = 12.0 Hz), 4.33 (d, 2H, *J* = 12.0 Hz), 3.47–3.43 (m, 2H), 2.63 (m, 1H), 2.30–2.04 (m, 2H), 1.78–0.99 (m, 10H), 0.77 (t, 3H, *J* = 6.9 Hz); ^13^C-NMR (CDCl_3_, 100 MHz) δ 174.8, 138.3, 133.4, 130.3, 128.3, 127.6 (2C), 73.1, 69.9, 67.6, 51.5, 47.2, 40.2, 33.0, 30.4, 29.7, 21.4, 14.1; LRMS (FAB) *m*/*z* 316 (M + H^+^); HRMS (FAB) calcd for C_20_H_30_NO_2_ (M + H^+^): 316.2277, found 316.2272.

#### 3.1.27. 2-Ethyl-*N*-(4-methoxybenzyl)-3-methylpent-4-enamide (**21’**)

To a solution of crotyl amide **21** (44 mg, 0.18 mmol) in toluene (2 mL), *i*PrMgCl (2.0 M in THF, 0.18 mL, 0.36 mmol) was added at reflux condition. After stirring at same temperature for 12 h, additional *i*PrMgCl (2.0 M in THF, 0.18 mL, 0.36 mmol) was added at same temperature. Reaction mixture was refluxed 5 h and cooled down to room temperature and quenched with brine and extracted with EtOAc. Organic layers were dried over MgSO_4_, filtered, evaporated, and purified by column chromatography on silica gel (EtOAc:*n*-hexane = 1:3) to afford lactam **21’** (18 mg, 36%) as an amorphous solid. IR (KBr) ν_max_ 3305, 2965, 1646, 1514, 1459 cm^−1^; ^1^H-NMR (CDCl_3_, 500 MHz) δ 7.18 (d, 2H, *J* = 7.6 Hz), 6.83 (d, 2H, *J* = 7.6 Hz), 5.79–5.72 (m, 1H), 5.59 (bs, 1H), 5.00–4.92 (m, 2H), 4.32 (d, 2H, *J* = 14 Hz), 3.77 (s, 3H), 2.40–2.34 (m, 1H), 1.83–1.77 (m, 1H), 1.71–1.48 (m, 3H), 1.11 (d, 3H, *J* = 5.5 Hz), 0.87 (t, 3H, *J* = 5.9 Hz); ^13^C-NMR (CDCl_3_, 125 MHz) δ 174.1, 158.9, 141.6, 130.5, 129.2, 114.2, 114.0, 55.3, 55.2, 42.8, 40.1, 23.0, 17.5, 12.2; LRMS (FAB) *m*/*z* 262 (M + H^+^); HRMS (FAB) calcd for C_16_H_24_NO_2_ (M + H^+^): 262.1807, found 262.1804.

### 3.2. Molecular Dynamics Simulations

In order to investigate and compare the structural stability of the reaction intermediate of the compounds, MD simulations were performed for 10 ns with GROMACS 5.1.5 using optimized potentials for liquid simulations—all-atom (OPLS-AA) [19]. The OPLS-AA force field was utilized to explain the interactions that involved lithium. Our ligand topology for the OPLS-AA force field was generated from the LigParGen server [20]. We have modified the parameter file for the oxygen–metal interaction since the original OPLS-AA force field did not provide it. The systems were solvated in the dodecahedron water box with tip3p water model, and then neutralized with the counter ions [21]. Each solvated system was energy minimized through steepest descent algorithm at 10,000 steps and maximum force lower than 1000 kJ/mol. In two equilibration steps, firstly the equilibration of each system was subjected to number of particles, volume, and temperature (NVT) equilibration at 100 ps at 300 K using Berendsen thermostat algorithm [22]. Secondly, the equilibration of each system was conducted using number of particles, pressure, and temperature (NPT) equilibration for 100 ps of 1 bar using the Parrinello–Rahman barostat [23]. The Linear Constraint Solver for Molecular Simulations (LINCS) algorithm [24] was used to restrain bond and heavy atom bonds. The settle algorithm [25] was utilized to restrict the geometry of water molecules. We calculated the long-range electrostatic interaction and a cut-off distance of 1.2 nm using the Particle Mesh Ewald (PME) algorithm [26]. MD simulations were conducted for 10 ns, saving the coordinate data for every 5 ps.

## 4. Conclusions

*i*PrMgCl is an advanced base of ACR. In addition, it is superior to the classic LHMDS/toluene condition when the sterically demanded substrate is subjected to ACR. The improvement of the aspect of reactions is derived from the relative stability of the desired conformation for ACR. MD simulations were carried out to investigate the structural stability of the reaction intermediates. The results of C-C-C-O dihedral angle analysis clearly showed that the structural stability of the intermediates was highly related to the portion of the product in the reaction. More detailed mechanistic investigations using other substrates, as well as their applications to the synthesis of bioactive molecules or natural products, will be discussed in due course.

## Figures and Tables

**Table 1:**
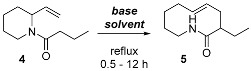
Screening of ACR conditions. LHMDS: lithium hexamethyldisilazane.

Entry	Base	Solvent	Result (%) ^a^
1	LHMDS	toluene	62
2	*i*PrMgCl	toluene	78
3	2-MesitylMgBr	toluene	53
4	*t*-BuMgBr	toluene	64
5	*n*-PropylMgBr	toluene	21
6	EtMgBr	toluene	31
7	NaHMDS	toluene	- ^b^
8	KHMDS	toluene	- ^b^
9	*i*PrMgCl	benzene	84
10	*i*PrMgCl	THF	23
11	*i*PrMgCl	*n*-decane	43
12	*i*PrMgCl	benzene ^c^	no reaction
13	*i*PrMgCl	benzene	81

^a^ Isolation yield; ^b^ Unidentified side product was obtained in 1 min.; ^c^ Reaction was carried out at the room temperature.

## Abbreviations

THF	Tetrahydrofuran
DIBAL	Diisobuylaluminum hydride
LHMDS	Lithium hexamethyldisilazane
LHMDS	Potassium hexamethyldisilazane
Boc	*tert*-Butyloxycarbonyl
TBDPS	*tert-*Butyldiphenylsilyl
TFA	Trifluoroacetic acid
DMF	Dimethylformamide
TBAF	Tetra-*n*-butylammonium fluoride
TBS	*tert*-Butyldimethylsilyl
Tf	Trifluoromethanesulfonate
TES	Triethylsilyl
EDCI	*N*-Ethyl-*N′*-(3-dimethylaminopropyl)carbodiimide hydrochloride
DMAP	4-Dimethylaminopyridine
Bn	Benzyl
TBAI	Tetra-*n*-butylammonium iodide
PMB	*para*-Methoxyl benzyl

## References

[1] Amirkia V., Heinrich M. (2014). Alkaloids as Drug leads—A Predictive Structural and Biodiversity-Based Analysis. Phytochem. Lett..

[2] Earley W.G., Jacobsen J.E., Madin A., Meier G.P., O’Donnell C.J., Oh T., Old D.W., Overman L.E., Sharp M.J. (2005). Aza-Cope Rearrangement—Mannich Cyclizations for the Formation of Complex Tricyclic Amines: Stereocontrolled Total Synthesis of (±)-Gelsemine. J. Am. Chem. Soc..

[3] Moon H., An H., Sim J., Kim K., Paek S., Suh Y. (2015). Efficient Strategy for the Stereoselective Synthesis of 2, 3-Disubstituted Benzo [A] Quinolizidine Alkaloids: Concise Synthesis of (−)-Protoemetinol. Tetrahedron Lett..

[4] Kretzschmar M., Hofmann F., Moock D., Schneider C. (2018). Intramolecular Aza-Diels–Alder Reactions of ortho-Quinone Methide Imines: Rapid, Catalytic, and Enantioselective Assembly of Benzannulated Quinolizidines. Angew. Chem. Int. Ed..

[5] Crossley S.W., Shenvi R.A. (2015). A Longitudinal Study of Alkaloid Synthesis Reveals Functional Group Interconversions as Bad Actors. Chem. Rev..

[6] Majumdar K.C., Bhattacharyya T., Chattopadhyay B., Sinha B. (2009). Recent Advances in the Aza-Claisen Rearrangement. Synthesis.

[7] Martín Castro A.M. (2004). Claisen Rearrangement Over the Past Nine Decades. Chem. Rev..

[8] Lee Y., Jung J., Kim S., Jung J., Paek S., Kim N., Chang D., Lee J., Suh Y. (2010). First Total Synthesis and Structural Confirmation of Fluvirucinine A_2_ Via an Iterative Ring Expansion Strategy. Org. Lett..

[9] Nubbemeyer U. (2005). Recent advances in charge-accelerated Aza-Claisen rearrangements. Natural Products Synthesis II.

[10] Jung J., Kim S., Suh Y. (2017). Advances in Aza-Claisen-Rearrangement-Induced Ring-Expansion Strategies. Asian J. Org. Chem..

[11] Tsunoda T., Sasaki O., Itô S. (1990). Aza-Claisen Rearrangement of Amide Enolates. Stereoselective Synthesis of 2, 3-Disubstituted Carboxamides. Tetrahedron Lett..

[12] Suh Y., Kim S., Jung J., Shin D., Min K., Koo B., Kim H. (1999). Asymmetric Total Synthesis of Fluvirucinine A_1_. Angew. Chem. Int. Ed..

[13] Paek S., Kim N., Shin D., Jung J., Jung J., Chang D., Moon H., Suh Y. (2010). A Concise Total Synthesis of (+)-Tetrabenazine and (+)-α-Dihydrotetrabenazine. Chem. Eur. J..

[14] Sonntag N.O. (1953). The Reactions of Aliphatic Acid Chlorides. Chem. Rev..

[15] Moon H., Yoon H., Lim C., Jang J., Yi J., Lee J., Lee J., Na Y., Son W., Kim S. (2018). Asymmetric Synthesis of (−)-6-Desmethyl-Fluvirucinine A1 Via Conformationally-Controlled Diastereoselective Lactam-Ring Expansions. Molecules.

[16] Jang J., Jung J., Ahn J., Sim J., Chang D., Kim D., Suh Y. (2012). Asymmetric Formal Synthesis of Schulzeines A and C. Org. Biomol. Chem..

[17] Arnold R.T., Searles S. (1949). A New Rearrangement of Allylic Esters. J. Am. Chem. Soc..

[18] Sato T., Yamazaki T., Nakanishi Y., Uenishi J., Ikeda M. (2002). A New Entry to 9-Azabicyclo [3.3. 1] Nonanes using Radical Translocation/Cyclisation Reactions of 2-(but-3-Ynyl)-1-(O-Iodobenzoyl) Piperidines. J. Chem. Soc. Perkin Trans. 1.

[19] Abraham M.J., Murtola T., Schulz R., Páll S., Smith J.C., Hess B., Lindahl E. (2015). GROMACS: High Performance Molecular Simulations through Multi-Level Parallelism from Laptops to Supercomputers. SoftwareX.

[20] Dodda L.S., Cabeza de Vaca I., Tirado-Rives J., Jorgensen W.L. (2017). LigParGen Web Server: An Automatic OPLS-AA Parameter Generator for Organic Ligands. Nucleic Acids Res..

[21] Jorgensen W.L., Chandrasekhar J., Madura J.D., Impey R.W., Klein M.L. (1983). Comparison of Simple Potential Functions for Simulating Liquid Water. J. Chem. Phys..

[22] Berendsen H.J., Postma J.V., van Gunsteren W.F., DiNola A., Haak J.R. (1984). Molecular Dynamics with Coupling to an External Bath. J. Chem. Phys..

[23] Parrinello M., Rahman A. (1981). Polymorphic Transitions in Single Crystals: A New Molecular Dynamics Method. J. Appl. Phys..

[24] Hess B., Bekker H., Berendsen H.J., Fraaije J.G. (1997). LINCS: A Linear Constraint Solver for Molecular Simulations. J. Comput. Chem..

[25] Miyamoto S., Kollman P.A. (1992). Settle: An Analytical Version of the SHAKE and RATTLE Algorithm for Rigid Water Models. J. Comput. Chem..

[26] Darden T., York D., Pedersen L. (1993). Particle Mesh Ewald: An N Log (N) Method for Ewald Sums in Large Systems. J. Chem. Phys..

